# Association of Genetic Polymorphisms with Ischemic Sudden Cardiac Death: A Comparative Case–Control Study in North-Western Transylvania (Romania)

**DOI:** 10.3390/biomedicines14030618

**Published:** 2026-03-10

**Authors:** Daniela Cristina Pavel (Mironescu), Costel Siserman, Mihaela Laura Vică Matei, Gheorghe Zsolt Nicula, Ștefana Bâlici, Bogdan-Alexandru Gheban, Ioana-Andreea Gheban-Roșca, Alexandra Șonfălean, Denisa Jurje, Denisa Lucian, Andrei Marușca, Daniel-Corneliu Leucuța, Horea-Vladi Matei

**Affiliations:** 1 Department of Forensic Medicine, Iuliu Hațieganu University of Medicine and Pharmacy, 400006 Cluj-Napoca, Romaniacvsiserman@gmail.com (C.S.); 2 Institute of Forensic Medicine, 400006 Cluj-Napoca, Romania; alesonfalean@gmail.com (A.Ș.); denisajurje97@yahoo.com (D.J.); pop_denisa2000@yahoo.com (D.L.); marusca.andrei@yahoo.com (A.M.); hmatei@umfcluj.ro (H.-V.M.); 3 Department of Cell and Molecular Biology, Faculty of Medicine, Iuliu Hațieganu University of Medicine and Pharmacy, 400349 Cluj-Napoca, Romania; gnicula@umfcluj.ro (G.Z.N.); sbalici@umfcluj.ro (Ș.B.); 4 Discipline of Fundamental Sciences, Faculty of Medical Assistance and Health Sciences, Iuliu Hațieganu University of Medicine and Pharmacy, 400023 Cluj-Napoca, Romania; ghebanbogdan@yahoo.com; 5 Department of Pathology, County Emergency Clinical Hospital, 400347 Cluj-Napoca, Romania; 6 Department of Medical Informatics and Biostatistics, Iuliu Hațieganu University of Medicine and Pharmacy, 400347 Cluj-Napoca, Romania; andreeiaioana.rosca@yahoo.com (I.-A.G.-R.); dleucuta@umfcluj.ro (D.-C.L.); 7 Department of Epidemiology, Clinical Hospital for Infectious Diseases, 400003 Cluj-Napoca, Romania

**Keywords:** sudden cardiac death, genetic predisposition, immunogenetics, *HLA-DRB1* typing, *HLA-DQB1* typing, *MYBPC3* gene, Single-Nucleotide Polymorphism (SNPs)

## Abstract

**Background/Objectives**: Ischemic sudden cardiac death (SCD) is a devastating event that often occurs in apparently healthy individuals. Genetic susceptibility may play a key role in the pathogenesis of such ischemic events. This study aimed to investigate the correlations between Human Leukocyte Antigen (HLA) alleles, genotypes, and haplotypes and SCD to identify potential risk factors. This study also investigated three Single-Nucleotide Polymorphisms (SNPs) in the *MYBPC3* gene and their association with SCD. **Methods**: We conducted an exploratory study between 2022 and 2024 in North-Western Transylvania (Romania) on 81 autopsy-confirmed SCD cases, compared with 162 controls for HLA typing, and with 96 controls for SNPs. HLA analysis of the *HLA-DRB1* and *HLA-DQB1* genes was performed using low-resolution SSP-PCR. The three SNPs in the *MYBPC3* gene: rs142317339 (C > T), rs148808089 (G > A), and rs11570076 (G > A) were performed using a Real-Time PCR System. **Results**: The HLA-DRB1*07 allele has reduced odds of SCD, after adjustment for age and sex, and the HLA-DRB1*08 allele showed a trend toward increased odds. No statistically significant associations were detected at the allele or genotype level for *HLA-DQB1*. Haplotype-based analyses further revealed that genetic susceptibility is driven predominantly by low-frequency protective haplotypes rather than by common risk haplotypes, with several combinations conferring strong or moderate protection (HLA-DRB1*07~HLA-DQB1*03, HLA-DRB1*07~HLA-DQB1*02, and HLA-DRB1*15~HLA-DQB1*05). No statistically significant association was found between the three SNPs studied in the two groups, and their frequencies were very low. **Conclusions**: Specific *HLA-DRB1* and *HLA-DQB1* alleles and haplotypes may be associated with protection against SCD, supporting a possible immunogenetic role in SCD and the identification of genetic risk markers.

## 1. Introduction

Sudden cardiac death (SCD) is a fatal event that occurs abruptly, often in individuals who appear clinically healthy. The medico-legal term “sudden cardiac death (SCD)” refers to deaths that occur unexpectedly and are not preceded by significant symptoms. This term excludes violent or traumatic deaths [1]. Definitions of SCD vary across countries, with time periods ranging from 1 to 48 h. The World Health Organization (WHO) defines SCD as a death occurring within 24 h of symptom onset. The Association for European Cardiovascular Pathology defines SCD as a natural death occurring within 6 h of the beginning of symptoms in an apparently healthy subject or in one whose disease is not severe enough to be expected to result in a fatal outcome. SCD in apparently healthy individuals, from newborns to adults, poses a significant challenge for medical examiners, law enforcement officers, and society due to the difficulty in interpreting epidemiological data and the variability in the definition of SCD [2]. Identifying the cause of SCD often requires collecting evidence through external examinations, crime scene investigations, and autopsies, as well as anamnesis and clinical data. However, in many cases, the cause of death remains undetermined even after thorough investigation [3].

Autopsy plays a crucial role in diagnosing sudden death, particularly in cases where the cause of death is not immediately apparent [4]. Here are the key roles of autopsy in such scenarios: determining cause of death by macroscopic evaluation, initial examination of the body and internal organs can reveal obvious causes of death, such as severe coronary artery disease, myocardial infarction, or other significant pathologies and microscopic analysis with histological examination of tissues can identify cellular and structural abnormalities that are not visible to the naked eye, such as myocarditis, fibrosis, or genetic conditions affecting the heart [5]. Autopsy plays a crucial role in sudden death, especially in discovering heart conditions such as hypertrophic cardiomyopathy, arrhythmogenic right ventricular cardiomyopathy, and congenital heart defects that could have led to death [6].

Molecular Autopsy with genetic testing in cases where the cause of death remains unexplained after traditional autopsy, molecular autopsy involves genetic testing to identify hereditary conditions like channelopathies (e.g., long QT syndrome) and cardiomyopathies [7]. A molecular autopsy is a specialized post-mortem examination that involves genetic testing to identify potential hereditary conditions that may have caused sudden death [8].

Toxicological analysis for substance detection in an autopsy includes toxicological testing to detect the presence of drugs, alcohol, or other toxic substances that could have contributed to sudden death [9]. Post-mortem imaging with advanced techniques, such as CT and MRI, can visualize internal structures and identify abnormalities that may not be detected by traditional autopsy methods [10,11]. The impact on family and public health of genetic counseling that identifies hereditary conditions through autopsy can lead to genetic counseling for family members, helping them understand their risk and take preventive measures [12]. Epidemiological data and autopsy findings contribute to public health knowledge, enhancing understanding of the causes of sudden death and informing the development of preventive strategies [2,13].

In summary, a molecular autopsy is a crucial tool in sudden death investigations, providing detailed genetic insights that can explain unexplained deaths, guide family risk assessments, and enhance preventive strategies [14,15]. Genetic factors, including the Human Leukocyte Antigen (HLA) system, have been implicated in susceptibility to cardiovascular disease and vascular inflammation [16]. Molecular autopsy is a vital tool for investigating sudden unexplained deaths (SUD), often caused by inherited arrhythmogenic diseases (AID). Genetic testing, especially using cost-effective Next-Generation Sequencing (NGS), helps identify pathogenic variants, enabling preventive measures for relatives [17]. Collaboration among forensic scientists, pathologists, cardiologists, and geneticists is crucial for accurate interpretation and clinical application [18,19].

In this context, genetic and immunological factors, particularly the HLA system, have been considered to play a significant role in susceptibility to acute cardiovascular events. HLA molecules, encoded by genes on chromosome 6, are involved in antigen presentation and the activation of the immune response [20]. Previous studies have demonstrated associations between specific HLA alleles and autoimmune, inflammatory, and cardiovascular diseases. The Major Histocompatibility Complex (MHC) consists of a complex of genes located on the short arm of chromosome 6 (locus 6p21) [21]. Specific proteins codified by these genes are involved in various immune system processes [20,22]. As a result, MHC is a highly polymorphic, multigenic, multiallelic complex with loci presenting linkage disequilibrium and codominant expression [23,24]. Because the HLA region has been shown to modulate the immune response, it has been identified as a major candidate region in genetic association studies of autoimmune diseases, including lupus erythematosus, psoriasis, multiple sclerosis, and autoimmune thyroiditis [24,25]. HLA class II genes present higher polymorphism compared to HLA class I genes, as they are found in both α and β chains [26,27]. The genetic mechanisms that generate HLA sequence polymorphisms include point mutations, certain recombination events, gene conversions, homologous and unequal crossing-over, and others [26,28]. The highest degree of polymorphism is observed in the variable domains of HLA molecules, which are involved in antigen binding [25,26], thereby altering the bound peptides that influence interactions with T cell receptors [25,26]. The varying frequencies of HLA alleles across populations can inform molecular autopsy by investigating HLA gene function and supporting the diagnosis of SCD [26]. Some studies conducted among the Transylvanian population of Romania have reported the frequency of HLA profiles and their association with specific pathological conditions [28].

Polymorphisms and variants in the *MYBPC3* gene, which encodes cardiac myosin-binding protein C, play a significant role in hypertrophic cardiomyopathy (HCM), a leading genetic cause of SCD characterized by ventricular hypertrophy, arrhythmia, and incomplete penetrance [29,30]. In Brazilian HCM patients, the benign *MYBPC3 p.Val158Met* polymorphism has been associated with severe left ventricular hypertrophy, with carriers showing a 13.5-fold increased risk, and combined sarcomeric variants exacerbating septum thickness and overall SCD susceptibility through reduced protein-tropomyosin interactions [31]. Pediatric studies from Asian cohorts reveal that *MYBPC3* pathogenic variants account for 24% of cases, correlating with a 10.2 odds ratio for sudden cardiac arrest, often presenting as the initial symptom in older children and highlighting limitations in current risk models for early intervention [32]. Furthermore, untargeted metabolomics in *MYBPC3* founder variant carriers identifies altered pathways in acylcarnitine, histidine, and purine metabolism linked to severe phenotypes, including malignant ventricular arrhythmias that heighten SCD risk, offering promising biomarkers for pathogenesis and personalized risk stratification [33].

This pilot study aims to investigate and generate preliminary data on the association between the *HLA-DRB1* and *HLA-DQB1* alleles, *HLA-DRB1* and *HLA-DQB1* genotypes, and *HLA-DRB1~HLA-DQB1* haplotypes with SCD in a case–control study of a Romanian cohort comprising 81 SCD cases and 162 controls. This study also investigated the distribution and potential association of three Single-Nucleotide Polymorphisms (SNPs) in the *MYBPC3* gene: rs142317339 (C > T), rs148808089 (G > A), and rs11570076 (G > A), in the same cohort of 81 SCD cases and 96 matched controls.

## 2. Materials and Methods

### 2.1. Study Population

The participants included consisted of two groups: a case group comprising 81 individuals who died from confirmed ischemic SCD, and a control group including 162 healthy individuals recruited from the general population. For each participant, *HLA-DRB1* and *HLA-DQB1* alleles were determined through genotyping. Allele, genotype, and haplotype frequencies were subsequently analyzed and compared between the case and control groups. For each identified genetic marker, the frequency in both groups was calculated, along with the odds ratio (OR), which serves as an estimate of the associated risk. Fisher’s exact test was then performed to evaluate statistical significance.

The study also investigated the distribution and potential association of three SNPs in the *MYBPC3* gene—rs142317339 (C > T), rs148808089 (G > A), and rs11570076 (G > A)—in a case group comprising 81 individuals who died from confirmed ischemic SCD and 96 healthy controls. ORs with 95% confidence intervals (CI) were computed for genetic associations.

### 2.2. Selection Criteria

This prospective autopsy-based exploratory study was conducted at the Institute of Forensic Medicine (IFM) in Cluj-Napoca, Romania. It included 81 cases of SCD examined between January 2022 and December 2024. The post-mortem interval was <24 h in all cases to limit autolysis; samples with visible degradation were excluded.

The primary aim was to elucidate the underlying causes of death. SCD was defined as a natural death within 24 h of symptom onset (WHO criteria), confirmed as CAD-related via autopsy (acute ischemia without other causes). Inclusion criteria comprised: confirmation of SCD attributable to acute myocardial ischemia secondary to coronary artery disease (CAD), substantiated by comprehensive autopsy findings and histopathological evidence of ischemic myocardial injury; availability of at least one significant coronary artery segment (left anterior descending, right coronary, or circumflex) with atherosclerotic plaque amenable to detailed histological analysis; and access to complete autopsy reports integrated with pertinent clinical data, including medical history and demographics, to facilitate robust clinicopathological correlation. As control groups, 162 individuals for HLA typing and 96 individuals for SNP analysis, without kinship among them, were enrolled in these studies and subjected to DNA paternity testing in the Molecular Biology Laboratory of the I.F.M. Cluj-Napoca. The control group consisted of individuals who, according to the questionnaire she completed and provided, had no history of cardiovascular disease at the time of the paternity tests.

Exclusion criteria included primary causes other than CAD (e.g., idiopathic hypertrophic or arrhythmogenic cardiomyopathy), traumatic/violent deaths, and chronic metabolic diseases (e.g., diabetes/hypertension). Exclusion criteria were stringently enforced to mitigate confounding variables, encompassing traumatic injuries, positive toxicological results (e.g., drug overdose or poisoning), or non-cardiac etiologies of death (e.g., pulmonary embolism, cerebrovascular accident, or organ failure unrelated to the heart). This rigorous selection yielded a homogeneous cohort representative of SCD due to CAD, thereby enabling targeted histopathological evaluation of coronary plaque morphology in the setting of acute myocardial ischemia while mitigating potential bias from confounding mortality factors.

This study was conducted in accordance with the Declaration of Helsinki and received ethical approval from the Ethics Committee of the Iuliu Hațieganu University of Medicine and Pharmacy Cluj-Napoca (Approval No. 288/15.11.2022). Informed consent was obtained from all subjects in the control group involved in the study and, for the case group, from relatives of deceased people.

### 2.3. Molecular and Genetic Analyses

#### 2.3.1. DNA Extraction

Genomic DNA was extracted from post-mortem peripheral blood (cases) and peripheral blood (controls) using standard protocols. From whole-blood samples collected from each subject in 3 mL sterile vacutainers with anticoagulant ethylenediaminetetraacetic acid (EDTA), DNA was extracted using Maxwell^®^ RSC Whole Blood (Promega, Madison, WI, USA), according to the manufacturer’s instructions, using the Maxwell RSC48 automatic extractor (Promega, Madison, WI, USA).

The DNA concentration and purity were analyzed using a NanoPhotometer P300 (Implen GmbH, Munich, Germany). Extracted DNA samples were considered sufficiently pure if the A260/A280 ratio was at least 1.8 ± 10%, and those that had a ratio lower than this were subjected to purification. For purification, when samples were not sufficiently pure, the EPICENTRE MasterPureTM Complete DNA and RNA Purification Kit (Illumina, Madison, WI, USA) was used. The obtained DNA was used for both HLA typing and/or SNP genotyping. The minimum DNA concentration to be used was 1 ng/μL for each well loaded for HLA typing, and for polymorphism analysis, it was between 1.5–5 ng/μL.

#### 2.3.2. HLA Typing

The *HLA-DRB1* and *HLA-DQB1* genes were typed using a molecular biology method. The DNA extracted from patients was amplified using a Single Specific Primer–Polymerase Chain Reaction (SSP-PCR). Amplification of each DNA sample was performed using the HLA-Fluogene typing kit (Inno-Train Diagnostik GmbH, Kronberg, Germany) according to the manufacturer’s instructions. These results were evaluated using a FluoVista Analyzer (Inno-Train Diagnostik GmbH, Kronberg, Germany).

#### 2.3.3. SNP Genotyping

SNP genotyping for rs142317339, rs148808089, and rs11570076 was performed via the TaqMan assay method. The rhAmp™ SNP Genotyping kit (Integrated DNA Technologies, Inc., Coralville, IA, USA) was used according to the manufacturer’s instructions with the QuantStudio™ 5 Real-Time PCR System (Thermo Fisher Scientific, Inc., Waltham, MA, USA).

### 2.4. Statistical Analysis

All individuals in the study (SCD group and the control group) were from North-Western Transylvania (Romania), and, according to 2021 census data, the region accounted for 13.24% of the Romanian population [34]. Differences between cases and controls were determined using Fisher’s exact test for alleles, genotypes, and haplotypes, and odds ratios (OR) with 95% confidence intervals (CI) were calculated. We computed corrected *p*-values for multiple comparisons using the Benjamini–Hochberg false discovery rate method. Multiple logistic regression models were fitted to predict SCD using HLA alleles identified as significant in the univariate analyses. The multiple logistic regression models were adjusted for age and sex. Goodness-of-fit was assessed using the Hosmer-Lemeshow test. Multicollinearity was assessed using the variance inflation factor. The linear relationship between continuous variables and the logit was checked, and the relationship between continuous variables and the logit was assessed using spline terms in a general additive model. Adherence to Hardy–Weinberg-equilibrium HWE was assessed in the control group (n = 162). Given the highly polymorphic, multi-allelic nature of these loci, a Monte Carlo Markov Chain (MCMC) permutation test with 10,000 iterations was performed to obtain exact *p*-values. Haplotype frequencies were counted, and their association with SCD was assessed with additive, dominant, and recessive effect models. Haplotype-specific odds ratios and 95% confidence intervals were estimated using additive logistic regression models adjusted for age and sex, with the most frequent haplotype serving as the reference. Extremely small ORs reflect quasi-complete separation arising from rare haplotypes absent or nearly absent among cases. There were no missing data in our dataset.

Statistical significance was defined at *p* < 0.05, and two-tailed *p*-values were considered. The haplotype analyses were performed using the haplo.stats R package version 1.9.3. All analyses were carried out in performed with R environment for statistical computing and graphics (R Foundation for Statistical Computing, Vienna, Austria), version 4.3.2. Population frequencies were extracted from gnomAD v4.1.0 (Broad Institute of MIT and Harvard, Cambridge, MA, USA) and ClinVar databases (National Center for Biotechnology Information, Bethesda, MD, USA).

## 3. Results

### 3.1. Characterization of the Study Groups

The study cohort comprised 243 participants, including 81 with SCD and 162 healthy controls. Overall, the demographic distribution revealed a predominance of male participants: 73.88% (n = 181) were male and 26.12% (n = 64) were female across the entire sample. This composition allowed for a comparative analysis of genetic and pathological factors between the affected and unaffected groups, focusing on allele frequencies and associated risk estimates. In the SCD group, participants ranged in age from 20 to 93 years, with a median age of 47 years (interquartile range [IQR]: 43–53 years). The group was notably skewed toward males, comprising 90.12% (n = 73) males and 9.88% (n = 8) females. Regarding residential background, 44.44% (n = 36) originated from rural areas, while 55.56% (n = 45) were from urban settings.

The control group consists of 146 men (90.12%) and 16 women (9.88%), with an average age of 46.9 years, all belonging to the same geographical areas of North-Western Transylvania. Additional demographic and clinical data were unavailable for the control group, precluding direct comparisons of these variables beyond the primary genetic analyses. No significant deviations from the Hardy–Weinberg equilibrium were detected in the control group for *HLA-DRB1* (*p* = 1) or *HLA-DQB1* (*p* = 1).

### 3.2. Characteristics of SCD Cases

Macroscopic autopsy findings in the SCD group revealed myocardial hypertrophy in 48.14% of cases, myocardiocoronarosclerosis in 88.89%, dilated cardiomyopathy in 39.5%, and generalized atherosclerosis in 39.51% of cases. Microscopic features were observed as follows: acute myocardial infarction in 45.68%, coronary atherosclerosis in 66.67%, myocardial fibrolipomatosis in 64.20%, hypertrophic cardiomyopathy in 25.93%, myocardiocoronarosclerosis in 38.27%, acute cerebral infarction in 4.94%, cerebral hemorrhage in 6.17%, pulmonary edema in 92.59%, pulmonary infarction in 7.41%, pulmonary emphysema in 77.78%, hepatic steatosis in 72.84%, hepatic cirrhosis in 2.47%, hepatitis in 3.70%, acute tubular necrosis in 70.37%, chronic pyelonephritis in 9.88%, and nephroangiosclerosis in 38.27%. Macroscopic and microscopic data are described in Table 1 and Table 2.

### 3.3. DNA Extraction

The DNA samples extracted from the control group did not require additional purification because they were obtained from whole blood, freshly collected in sterile vacutainers containing EDTA. Only 6 extracted DNA samples (~7.41%) of the 81 samples belonging to the case group (from SCDs) required additional purification. After DNA extraction and purification, all samples could be successfully genotyped, both by HLA typing and by polymorphism analysis, because the DNA quantity was sufficient and its quality was adequate.

### 3.4. HLA Typing Data

Several *HLA-DRB1* alleles showed differences in frequency between the SCD and control groups. The HLA-DRB1*07 allele was found less frequently in the SCD group compared to the control group (3.09% vs. 9.88%), which suggests that individuals carrying HLA-DRB1*07 have significantly reduced odds of SCD (OR = 0.29, 95% CI 0.11–0.76, *p* = 0.008). In contrast, the HLA-DRB1*08 allele, which is more frequent in SCD than in the control group (3.70% vs. 0.93%), suggests higher odds of SCD, albeit close to the significance level (OR = 4.12, 95% CI 1.02–16.67, *p* = 0.066). After Benjamini–Hochberg false discovery rate correction, the results lost their statistical significance, for the allele HLA-DRB1*07, the *p*-value being 0.104. Some alleles were more frequent in the SCD group, respectively in the control group, but did not reach statistical significance.

Across all five evaluated *HLA-DQB1* allele groups (*02, *03, *04, *05, *06), none showed a statistically significant association with SCD.

The distribution of *HLA-DRB1* and *HLA-DQB1* allele frequencies within the SCD cases and controls is presented in detail in Table 3, Table 4 and Table 5.

For the *HLA-DRB1* alleles that were statistically significant before correction, we built multivariate logistic regression models, adjusted for age and sex (Table 4). After adjustment, HLA-DRB1*07 remained statistically significant, being associated with reduced odds of SCD. After adjustment, the HLA-DRB1*08 got closer to the level of significance (*p* = 0.051).

The most frequent *HLA-DRB1~HLA-DQB1* haplotypes in the SCD and control groups are presented in Table 6.

Haplotype-based analysis demonstrated a significant global association between *HLA-DRB1~HLA-DQB1* haplotypes and SCD presence (global *p* = 0.0055). Given low haplotype frequencies and model stability considerations, association results are reported using an additive haplotype effect model. Three low-frequency haplotypes (DRB1*07~DQB1*03, DRB1*15~DQB1*05, and DRB1*07~DQB1*02) showed protective associations (Table 7). No haplotype demonstrated a strong risk-increasing effect. Then, we performed the same analysis, adjusting for age and sex. The results remained statistically significant (global *p* = 0.0063), indicating the same protective effect and the robustness of our findings.

Two haplotypes, which had low frequencies DRB1*07~DQB1*03 (frequency 2.1%) and DRB1*15~DQB1*05 (frequency 2.5%), with absolute counts of 10 and 12, showed protective effects, being observed almost exclusively among controls, resulting in odds ratios approaching zero (Table 8). A third haplotype, DRB1*07~DQB1*02 (frequency 5.3%), showed a protective effect (OR = 0.33, 95% CI 0.10–1.07), indicating approximately a 67% reduction in SCD odds. However, this association did not reach statistical significance after covariate adjustment. No common haplotype demonstrated a strong risk-increasing effect.

### 3.5. SNP Genotype Distributions

The rs142317339 was detected only in 2 heterozygous cases, absent in controls (allele frequency 1.23% vs. 0%, *p* = 0.16). The rs148808089 was monomorphic in both cases and controls. The rs11570076 showed a slight, non-significant increase in the allele A frequency in cases compared to controls (11.7% vs. 8.8%, OR = 1.38, 95% CI 0.72–2.63, *p* = 0.33). Table 9 presents the distribution of selected SNPs across both groups.

Population database analysis revealed rs142317339 as an extremely rare variant (<0.001% in gnomAD and ClinVar). The rs11570076 is a missense variant (p.Arg382Trp) with a low-moderate population frequency (~1.4%). Functional prediction tools and literature suggest a possible impact on MYBPC3 protein structure and sarcomere function, though the clinical significance remains uncertain.

## 4. Discussion

The present pilot study explored potential genetic evidence for associations between specific *HLA-DRB1* and *HLA-DQB1* alleles, genotypes, and haplotypes and the risk of SCD in a Romanian population from North-Western Transylvania. We identified specific HLA variants that were either enriched or depleted among SCD cases, indicating a possible immunogenetic contribution to fatal cardiac events.

At the single-allele level, HLA-DRB1*07 was underrepresented among SCD cases and remained independently associated with reduced odds of SCD after adjustment for age and sex, whereas HLA-DRB1*08 showed a trend toward increased risk in age- and sex-adjusted models. No *HLA-DQB1* allele demonstrated an independent association with SCD. These allele-level findings are mirrored by the haplotype analysis, in which we identified a significant association between *HLA-DRB1~HLA–DQB1* haplotypes and SCD susceptibility, due mainly to low-frequency protective haplotypes rather than common risk haplotypes. In age- and sex-adjusted additive models, DRB1*07–DQB1*03 and DRB1*15–DQB1*05 showed near-complete protection, while DRB1*07–DQB1*02 demonstrated a moderate protective effect. No haplotype exhibited a strong risk-increasing association.

This study also reports the distribution of three *MYBPC3* SNPs in Romanian SCD cases and matched controls, which is very low, and no significant associations were found.

HLA-DRB1*08 and HLA-DRB1*09 have been previously associated with heightened inflammatory responses and autoimmune disease [35], supporting the concept that immune activation contributes to plaque vulnerability and rupture.

We identified three *HLA-DRB1-DQB1* haplotypes with protective associations. Notably, the HLA-DRB1*07-DQB1*03 and HLA-DRB1*15-DQB1*05 haplotypes occurred at low frequencies within our cohort (absolute counts of n = 10 and n = 12, respectively). While the negative Hap-Scores suggest a protective biological effect, we interpret these findings with caution. Rare haplotypes are susceptible to statistical instability and ‘winner’s curse’ effects in smaller cohorts; therefore, these associations should be considered hypothesis-generating until validated in larger, multi-center international datasets. In contrast, the protective haplotypes (HLA-DRB1*07~HLA-DQB1*03, HLA-DRB1*07~HLA-DQB1*02, and HLA-DRB1*15~HLA-DQB1*05) promote a more balanced immune response, potentially reducing inflammatory damage to the fibrous cap and limiting progression of the necrotic core. Taken together, the findings suggest that SCD associated with coronary atherothrombosis may be partially mediated by genetically determined immune-inflammatory pathways linked to specific *HLA-DRB1~HLA-DQB1* haplotypes.

Although the inferential analysis suggested a possible statistical significance of risk or protection of some HLA genetic markers, the statistical significance was preserved after adjustment only for the *HLA*-DRB1*07 allele as a possible protective factor, respectively for the haplotypes HLA-DRB1*07~HLA-DQB1*03, HLA-DRB1*07~HLA-DQB1*02, and HLA-DRB1*15~HLA-DQB1*05 as a possible protective factor.

These findings align with the hypothesis that HLA class II alleles modulate immune responses contributing to cardiovascular pathology, potentially through antigen presentation that exacerbates inflammation or autoimmunity in CAD, a primary underlying cause of SCD in our cohort. In contrast, the protective haplotypes (HLA-DRB1*07~HLA-DQB1*03, HLA-DRB1*07~HLA-DQB1*02, and HLA-DRB1*15~HLA-DQB1*05) may be associated with a more balanced immune response, potentially linked to reduced inflammatory damage to the fibrous cap and limited progression of the necrotic core. Taken together, the findings suggest that SCD associated with coronary atherothrombosis may be partially influenced by genetically determined immune-inflammatory pathways potentially linked to specific HLA-DRB1~HLA-DQB1 haplotypes. Although immunogenetic mechanisms may offer a possible explanatory framework, these findings reflect statistical associations rather than causality. Replication in larger populations and functional validation are required to clarify their biological relevance.

Comparisons with existing literature reveal both consistencies and population-specific variations in HLA associations with cardiovascular diseases. For instance, HLA-DRB1*01 has been identified as a risk factor for acute myocardial infarction (AMI) in a Swedish cohort, in which HLA-DRB1*01:01 was associated with an elevated OR, independent of traditional risk factors [36]. Besides this, in heart failure patients, allele frequency analyses have revealed variations in the distributions of *HLA-DRB1* and *HLA-DQB1*, with specific haplotypes, such as HLA-DRB1*03:01~HLA-DQB1*02:01, being common, potentially influencing immune-mediated myocardial damage [37]. This observation does not align with our findings of HLA-DRB1*08 enrichment in SCD, suggesting a different allele with a broader role in ischemic events that lead to fatal arrhythmia.

Other studies on diastolic dysfunction in sarcoidosis have identified specific *HLA-DRB1* alleles, such as HLA-DRB1*14, as linked to cardiac involvement, albeit in a different context [38]. However, HLA-DRB1*15:01 has been associated with increased AMI risk in other populations, highlighting potential ethnic differences [39,40,41,42]. Conversely, the HLA-DRB1*15 allele, known for its anti-inflammatory regulatory effects, was observed more frequently in our control group, without a significant difference from the SCD group. Still, it is present within the HLA-DRB1*15~HLA-DQB1*05 haplotype, which has been shown to confer a protective effect in our cohort. These findings reinforce the concept that immune imbalance contributes to SCD pathogenesis.

Regarding protective alleles, we observed the depletion of HLA-DRB1*07 in our SCD group. Our findings suggest HLA-DRB1*07 may confer resistance to SCD, possibly by altering cytokine profiles or T-cell responses that mitigate plaque instability.

Although we did not observe significant protective or risk associations of *HLA-DQB1* alleles with SCD, there is evidence for idiopathic dilated cardiomyopathy (IDCM) in the Han of Northern China, where HLA-DQB1*03:03 (part of the *03 cluster) has been linked to susceptibility [40]. The HLA-DQB1*03 allele showed a non-significant trend towards a higher frequency in our SCD group, which may reflect allelic subgroups or sample size limitations.

Male sex was strongly associated with SCD in this study, consistent with the global epidemiology, potentially attributable to hormonal modulation of inflammation and endothelial dysfunction. Notably, a protective role for HLA-DRB1*12:02:01 in CAD was reported in southern Han Chinese women, suggesting gender-HLA interactions that warrant further exploration in SCD [40]. In our cohort, the lack of significant HLA-DRB1*12 associations may stem from ethnic differences, as allele frequencies vary globally; for example, HLA-DRB1*12 is less common in European-derived populations compared to East Asians [40].

The pathological findings in our SCD cases include high rates of coronary atherosclerosis (66.67%), myocardial hypertrophy (48.14%), and pulmonary edema (92.59%), which underscore the atherosclerotic basis of SCD. HLA class II alleles likely contribute via immune dysregulation, as evidenced by associations with lipoprotein(a) [Lp(a)] levels, a CAD risk factor. Although no direct Lp(a)-HLA linkage was found in early-onset CAD [43], other studies link *HLA-DR* genotypes to Lp(a) in autoimmune-like atherosclerosis [44,45].

Broader immunogenetic contexts support these associations. HLA class II variability influences susceptibility to autoimmune diseases with cardiovascular overlap, such as rheumatoid arthritis and systemic lupus erythematosus, in which shared epitopes, such as HLA-DRB1*04, increase risk [46,47,48,49]. Population studies in diverse ethnicities [41,42] highlight allele diversity, emphasizing the need for region-specific data, as in our Transylvanian cohort.

Similarly, in type 1 diabetes, specific HLA class II haplotypes, such as HLA-DRB1*10:01~HLA-DQB1*05:01, have been independently associated with cardiovascular events and death, indicating a broader immunogenetic influence on vascular pathology [50]. Coronary atherosclerosis is a multifactorial disease in which the interaction between genetic susceptibility and immune activation drives not only plaque formation but also its progression toward instability and rupture. Current immunogenetic models emphasize at least three functional axes that converge toward a pro-rupture phenotype: chronic activation of innate immunity and inflammatory signaling (e.g., polymorphisms in *TLRs*, *NLRP3*, *IL1B*) that maintain a persistent proinflammatory milieu within the fibrous cap; dysregulation of adaptive immunity—predominance of Th1/Th17 responses combined with impaired T-regulatory cell activity (genetically influenced through HLA variants, *IL23R*, *FOXP3*/*CTLA4*)—promoting sustained recruitment and activation of macrophages and lymphocytes within the lipid core; and genes regulating lipid metabolism and oxidative stress (*APOE*, *LDLR*, *PCSK9*, *PON1*, *SOD2*), which alter LDL quantity and quality (oxidized LDL) and thus amplify local immune activation [51,52]. From a pathological perspective, the combination of a genetically driven “high-inflammatory phenotype” with an “atherogenic lipid profile” tends to produce plaques with large necrotic cores, intense inflammatory infiltrates, and thin fibrous caps—hallmark features associated with plaque instability and thrombogenic potential [51,52].

The absence of strong genotype associations in our cohort may reflect population-specific haplotype diversity or limited power to detect rare combinations, as evidenced by the weak associations between *HLA-DRB1* and *HLA-DQB1* and acute myocardial infarction in other populations [43], underscoring the need for haplotype-resolved analyses to elucidate epistatic interactions in SCD etiology.

The present study demonstrates that the analyzed *MYBPC3* variants are either rare (rs142317339), monomorphic (rs148808089), or show only a modest, non-significant frequency difference (rs11570076) in Romanian SCD cases versus controls. The three *MYBPC3* SNPs were selected as exploratory variants based on previous reports of associations with cardiomyopathies in other populations, according to OMIM, an online catalog of human genes and genetic disorders [53]. Although there is no evidence regarding the direct association between these polymorphisms and SCD, we attempted to study whether their involvement in cardiomyopathies could lead to SCD. The rs142317339, identified in only 2 heterozygous cases (samples 64 and 66), corroborates global population data indicating extreme rarity, but rs11570076, although more frequent (~1.4%), did not show a significant association with SCD (OR = 1.37, 95% CI 0.69–2.73, *p* = 0.3837), suggesting a limited effect size in this cohort.

The lack of significant association may reflect the small sample size, the polygenic nature of SCD, and the potential influence of environmental or comorbid factors. Nonetheless, the presence of rare variants in *MYBPC3* underscores the need for functional studies to assess pathogenicity, particularly for variants such as rs11570076, which have known missense effects. Comparison with population databases confirms the rarity of rs142317339 and the modest frequency of rs11570076, reinforcing the importance of integrating population genetics in the interpretation of candidate SCD variants.

Limitations include the relatively modest sample size, from one region in Romania. There is no exact age match between the case and control groups, as the subjects presenting for paternity testing are generally younger. However, the mean ages between the two study groups are very close. On the other hand, the male predominance in the case group (that is equal to the control group) is explained by the higher frequency of cardiovascular disease in men compared to women. Nevertheless, we performed multivariate analyses, adjusting for age and sex, to address these issues. The control group lacked clinical data, precluding adjustments for subclinical CAD. ‘Healthy’ controls were defined as individuals without self-reported cardiovascular events or family history of SCD; however, subclinical disease was not screened. Thus, the control group does not constitute a clinically characterized cardiovascular reference population, and this limits the power of causal inference. Autopsy-based selection may be biased toward severe cases [54], and the absence of typing at certain loci limits comprehensive haplotype analysis. Nonetheless, our low-resolution typing and adjusted regression models strengthen inferences. Another limitation is the focus on only three SNPs within a single gene.

Our study also has several strengths. The findings were concordant between score-based haplotype tests and regression-based haplotype models, and even after adjusting for confounders, strengthening confidence in the robustness of the associations. Also, to our knowledge, this is the first study to characterize the *MYPPC3* in the Romanian population.

Our scientific work is an exploratory, hypothesis-generating study examining several potential genetic markers associated with sudden death. Future directions involve larger, multi-ethnic cohorts to validate these associations and explore haplotypes. Functional studies, such as peptide-binding assays or larger multicenter cohorts, could elucidate how risk alleles present cardiac antigens. Moreover, next-generation sequencing or larger multicenter cohorts would be needed to evaluate the role of *MYBPC3* in SCD further. Integrating HLA with genome-wide association studies (GWAS) for CAD may reveal polygenic interactions, aiding risk stratification and personalized prevention in high-risk populations. Future studies should prioritize larger samples, matched controls with clinical assessments, high-resolution typing, adjusted models, and functional assays to clarify mechanisms. Regional studies could enhance predictive value. This study highlights challenges in SCD genetics and calls for rigorous replication.

## 5. Conclusions

In this case–control study conducted in North-Western Transylvania, Romania, our results show that variation within the HLA class II region, particularly at the *HLA-DRB1* locus and within specific *HLA-DRB1~HLA-DQB1* haplotypes, is associated with the susceptibility to sudden cardiac death. Across both allele-level and haplotype-level analyses, HLA-DRB1*07 consistently emerged as a protective factor, while HLA-DRB1*08 showed a trend toward increased odds. Haplotype-based analyses further revealed that genetic susceptibility is driven predominantly by low-frequency protective haplotypes rather than by common risk haplotypes, with several *HLA-DRB1~HLA-DQB1* combinations conferring strong or moderate protection (HLA-DRB1*07~HLA-DQB1*03, HLA-DRB1*07~HLA-DQB1*02, and HLA-DRB1*15~HLA-DQB1*05).

This study also reports the distribution of three *MYBPC3* SNPs in Romanian SCD cases and matched controls. The rs142317339 is extremely rare, rs148808089 is monomorphic, and rs11570076 shows a non-significant trend towards higher frequency in cases. No statistically significant associations were observed, underscoring the rarity of these *MYBPC3* variants and their limited detectable effect in SCD.

## Figures and Tables

**Table 1:** Macroscopic characteristics of sudden cardiac death (SCD) cases.

Macroscopic	No. of Cases	% of Total SCD Cases
Myocardial hypertrophy	39	48.14
Myocardiocoronarosclerosis	72	88.89
Dilated cardiomyopathy	32	39.51
Generalized atherosclerosis	32	39.51

SCD, sudden cardiac death.

**Table 2:** Microscopic characteristics of SCD cases.

Microscopic	No. of Cases	% of Total SCD Cases
Acute myocardial infarction	37	45.68
Coronary atherosclerosis	54	66.67
Myocardial fibrolipomatosis	52	64.20
Hypertrophic cardiopathy	21	25.93
Myocardiocoronarosclerosis	31	38.27
Acute cerebral infarction	4	4.94
Cerebral hemorrhage	5	6.17
Pulmonary edema	75	92.59
Pulmonary infarction	6	7.41
Pulmonary emphysema	63	77.78
Hepatic steatosis	59	72.84
Hepatic cirrhosis	2	2.47
Hepatitis	3	3.70
Acute tubular necrosis	57	70.37
Chronic pielonephritis	8	9.88
Nephroangiosclerosis	31	38.27

SCD, sudden cardiac death.

**Table 3:** *HLA-DRB1* Allele Odds Ratio/Risk Ratio for SCD.

*HLA-DRB1* Alleles	SCD Group (n = 81)/162 *	Control Group (n = 162)/324 **	OR (95% CI)	*p Adjusted* ^#^	*p*
* *01*	17 (10.49)	23 (7.1)	1.53 (95% CI 0.8–2.96)	0.735	0.199
* *03*	18 (11.11)	40 (12.35)	0.89 (95% CI 0.49–1.6)	0.897	0.692
* *04*	13 (8.02)	27 (8.33)	0.96 (95% CI 0.48–1.91)	0.983	0.907
* ***07***	5 (3.09)	32 (9.88)	0.29 (95% CI 0.11–0.76)	0.104	**0.008**
* *08*	6 (3.7)	3 (0.93)	4.12 (95% CI 1.02–16.67)	0.429	0.066
* *09*	3 (1.85)	2 (0.62)	3.04 (95% CI 0.5–18.36)	0.735	0.339
* *10*	3 (1.85)	9 (2.78)	0.66 (95% CI 0.18–2.47)	0.897	0.759
* *11*	40 (24.69)	70 (21.6)	1.19 (95% CI 0.76–1.86)	0.823	0.443
* *12*	3 (1.85)	8 (2.47)	0.75 (95% CI 0.2–2.85)	0.897	0.759
* *13*	15 (9.26)	33 (10.19)	0.9 (95% CI 0.47–1.71)	0.897	0.747
* *14*	10 (6.17)	20 (6.17)	1 (95% CI 0.46–2.19)	1.000	1
* *15*	13 (8.02)	35 (10.8)	0.72 (95% CI 0.37–1.4)	0.735	0.333
* *16*	16 (9.88)	22 (6.79)	1.5 (95% CI 0.77–2.95)	0.735	0.232

HLA, human leukocyte antigen; SCD, sudden cardiac death; n, number of persons; *^,^** total number of alleles in the analyzed group; OR, odds ratio; CI, confidence interval; *p* value < 0.05 (highlighted in bold) was considered; ^#^**,** Benjamini–Hochberg false discovery rate corrected *p*-values for multiple comparisons.

**Table 4:** Multiple logistic regression models predicting SCD adjusting for age and sex.

*HLA-DRB1*	*OR Adjusted*	(95% CI)	*p*
*07	0.29	(0.1–0.7)	0.012
*08	4.03	(1.04–19.38)	0.051

HLA, human leukocyte antigen; OR, odds ratio; CI, confidence interval.

**Table 5:** *HLA-DQB1* Allele Odds Ratio/Risk Ratio for SCD.

*HLA-DQB1* Alleles	SCD Group (n = 81)/162 *	Control Group (n = 162)/324 **	OR (95% CI)	*p-Adjusted* ^#^	*p*
*02	26 (16.05)	64 (19.75)	0.78 (95% CI 0.47–1.28)	0.805	0.322
*03	63 (38.89)	120 (37.04)	1.08 (95% CI 0.73–1.59)	1	0.691
*04	4 (2.47)	3 (0.93)	2.71 (95% CI 0.6–12.25)	0.805	0.229
*05	43 (26.54)	85 (26.23)	1.02 (95% CI 0.66–1.56)	1	0.942
*06	26 (16.05)	52 (16.05)	1 (95% CI 0.6–1.67)	1	1

HLA, human leukocyte antigen; SCD, sudden cardiac death; n, number of persons; *^,^** total number of alleles in the analyzed group; OR, odds ratio; CI, confidence interval; ^#^**,** Benjamini–Hochberg false discovery rate corrected *p*-values for multiple comparisons.

**Table 6:** Frequency distribution of the most prevalent *HLA-DRB1~HLA-DQB1* haplotypes in the *SCD* group and the control group.

SCD			Control		
*HLA-DRB1*	*HLA-DQB1*	Haplotype Frequency	*HLA-DRB1*	*HLA-DQB1*	Haplotype Frequency
*11	*03	0.228	*11	*03	0.210
*03	*02	0.111	*03	*02	0.123
*01	*05	0.092	*13	*06	0.083
*16	*05	0.086	*04	*03	0.077
*15	*06	0.080	*01	*05	0.071
*04	*03	0.062	*15	*06	0.071
*13	*06	0.062	*07	*02	0.068
*14	*05	0.056	*16	*05	0.065
*08	*03	0.037	*14	*05	0.058
*07	*02	0.025	*15	*05	0.037

HLA, human leukocyte antigen; SCD, sudden cardiac death.

**Table 7:** Haplotypes association with SCD, in unadjusted and adjusted for sex and age, using the additive model.

Rank	Haplotype	Frequency (%)	Unadjusted Score	Adjusted Score	Unadjusted *p*	Adjusted *p*
1	DRB1*07~DQB1*03	10 (2.1)	−2.21	−2.19	0.0272	0.0285
2	DRB1*15~DQB1*05	12 (2.5)	−2.16	−2.15	0.0307	0.0312
3	DRB1*07~DQB1*02	26 (5.3)	−1.97	−1.98	0.0493	0.0476

**Table 8:** Association of *HLA-DRB1~HLA-DQB1* Haplotypes with Disease Status Using Age- and Sex-Adjusted Additive Logistic Regression.

*HLA-DRB1*	*HLA-DQB1*	Frequency	OR (95% CI)	*p*
*07	*03	0.021	<0.05	0.0000
**	**	0.038	6.50 (1.92–22.10)	0.0030
*07	*02	0.053	0.33 (0.10–1.07)	0.0667
*13	*06	0.076	0.54 (0.21–1.35)	0.1890
*04	*03	0.072	0.59 (0.24–1.46)	0.2570
*08	*04	0.010	3.21 (0.23–45.20)	0.3880
*10	*05	0.025	0.52 (0.12–2.36)	0.4010
*09	*03	0.010	2.09 (0.28–15.40)	0.4710
*03	*02	0.119	0.78 (0.38–1.59)	0.5010
*12	*03	0.023	0.58 (0.12–2.88)	0.5030
*14	*05	0.057	0.76 (0.30–1.88)	0.5490
*04	*02	0.010	1.68 (0.22–12.50)	0.6140
*16	*05	0.072	1.24 (0.53–2.87)	0.6200
*15	*06	0.074	1.10 (0.46–2.64)	0.8350
*13	*03	0.020	0.88 (0.21–3.65)	0.8590
*01	*05	0.078	0.99 (0.44–2.19)	0.9740
*15	*05	0.025	<0.05	

HLA, human leukocyte antigen; OR, odds ratio; CI, confidence interval; **, rare haplotypes.

**Table 9:** Distribution of selected SNPs (Single-Nucleotide Polymorphisms) allele group frequencies in the study cohort.

SNP	Genotype	SCD Group (n = 81)	Control Group (n = 96)	Allele Frequencies (%)		*p* (Fisher)	OR (95% CI)	Interpretation
		No. (%)	No. (%)	SCD Group	Control Group			
rs142317339 (C > T)	C/C	79 (97.5%)	96 (100%)	C: 98.77%	C: 100%	0.2087	—	Rare variant, no significant association
	C/T	2 (2.5%)	0 (0%)	T: 1.23%	T: 0%			
	T/T	0 (0%)	0 (0%)					
rs148808089 (G > A)	G/G	81 (100%)	96 (100%)	G: 100%	G: 100%	—	—	Monomorphic variant
	G/A	0 (0%)	0 (0%)	A: 0%	A: 0%			
	A/A	0 (0%)	0 (0%)					
rs11570076 (G > A)	G/G	69 (85.2%)	86 (89.6%)	G: 88.27%	G: 91.15%	0.3837	1.37 (0.69–2.73)	Slight, nonsignificant tendency
	G/A	5 (6.2%)	3 (3.1%)	A: 11.73%	A: 8.85%			
	A/A	7 (8.6%)	7 (7.3%)					

SNP, Single-Nucleotide Polymorphism; SCD, sudden cardiac death; OR, odds ratio; CI, confidence interval.

## Data Availability

The original contributions presented in the study are included in the article. Any further inquiries may be directed to the corresponding author.

## Abbreviations

The following abbreviations are used in this manuscript:

CAD	Coronary Artery Disease
HLA	Human Leukocyte Antigen
IFM	Institute of Forensic Medicine
MHC	Major Histocompatibility Complex
SCD	Sudden Cardiac Death
SNP	Single-Nucleotide Polymorphism
